# Left Ventricular Function in a Large Cohort of Pseudoxanthoma Elasticum Patients

**DOI:** 10.1371/journal.pone.0090364

**Published:** 2014-03-20

**Authors:** Loïc Bière, Erwan Donal, Gwenola Terrien, Alain Furber, Ludovic Martin, Fabrice Prunier

**Affiliations:** 1 L'UNAM Université, Angers, France; 2 Université Angers, Laboratoire Cardioprotection, Remodelage et Thrombose, CHU Angers, Service de Cardiologie, Angers, France; 3 Service de Cardiologie et CIC-IT 804, CHU de Rennes & Laboratoire Traitement du Signal et de l'Image, INSERM U1099, Rennes, France; 4 CHU Angers, Centre de consultation PXE, Angers, France; 5 Université Angers, Biologie Neurovasculaire et Mitochondriale Intégrée, UMR INSERM-1083 CNRS-6214, Angers, France; Merck & Co., United States of America

## Abstract

**Objective:**

Pseudoxanthoma elasticum (PXE) is a rare autosomal recessive disorder characterized by the mineralization and fragmentation of elastic fibers in the skin, retina, and vascular walls. While there is no doubt that peripheral arterial disease is associated with PXE, several other cardiac complications have been linked with PXE, mainly based on case reports. It remains unclear, whether cardiac systolic or diastolic function impairment is a common complication of PXE.

**Methods:**

This study conducted systematic assessment of left ventricular systolic and diastolic function via standard echocardiography and two-dimensional strain imaging, in a large cohort of asymptomatic PXE patients (n = 75) and matched healthy controls (n = 30).

**Results:**

PXE and controls did not differ in terms of any of the diastolic parameters tested: E-wave (82±17 cm/s *vs.* 82±13 cm/s, p = 0.890), E deceleration time (191.7±55.6 ms *vs.* 190.0±35.9 ms, p = 0.879), and E/Em ratio (7.1±2.3 *vs.* 7.0±1.8, p = 0.829). In addition, no significant differences were observed between PXE and control in terms of left ventricular volumes and ejection fraction, as well as global, basal, mid, and apex longitudinal strains.

**Conclusions:**

These findings revealed that preclinical cardiac dysfunction is uncommon in a large population of asymptomatic PXE patients.

## Introduction

Pseudoxanthoma elasticum (PXE) is a rare autosomal recessive disorder characterized by the mineralization and fragmentation of elastic fibers in the skin, retina, and vascular walls [1]. PXE is diagnosed based on the combined dermatological features of yellowish papules and loss of skin elasticity on the main flexural areas, along with eye lesions consisting of angioid streaks. Peripheral arterial disease is commonly associated with PXE [2]. Although few reports of PXE patient autopsies revealed direct involvement of the myocardium with endocardial lesions, characterized by degenerated elastic fibers with calcification in the subendocardium [3], it remains unclear whether cardiac systolic or diastolic function impairment is commonly found in PXE [4]–[6]. This study conducted systematic assessment of left ventricular (LV) systolic and diastolic function using standard echocardiography and two-dimensional strain imaging, a sensitive tool used to detect early systolic dysfunction in preclinical disease [7], in a large cohort of PXE patients and matched healthy controls.

## Methods

This study's protocol was approved by the institutional ethics committee at the University Hospital of Angers and the study was conducted in accordance with the Helsinki Declaration and French regulations. All patients provided written informed consent prior to inclusion. The study was registered at ClinicalTrials.gov (Identifier: NCT01446380).

### PXE cohort

A total of 81 adult PXE patients were prospectively included in the referral PXE consultation center at the University Hospital of Angers in France between 2008 and 2013. PXE diagnosis was based on the following defined criteria: clinically indicative skin changes, angioid streaks, and histological demonstration of fragmented and calcified elastic fibers on skin biopsy [8].

For each patient, we routinely assessed body height and weight, blood pressure, medical history, physical examination, and 12-lead electrocardiography (MAC5500 resting ECG analysis system, GE Healthcare, Freiburg, Germany). The following cardiovascular risk factors were recorded for all patients: smoking habits, hypertension (*i.e.*, systolic/diastolic blood pressure >140/90 mmHg in office measurements or antihypertensive medication use), diabetes (fasting glucose ≥1.25 g/L or glucose-lowering medication use), and hyperlipemia (low-density lipoprotein cholesterol <1.3 g/L, high-density lipoprotein cholesterol <0.40 g/L, triglyceridemia >1.5 g/L or lipid-lowering medication use). Blood glucose and lipids analysis were set on dedicated blood samples. Coronary artery disease was ruled out with treadmill test and single-photon emission computed tomography [6].

A total of six PXE patients were excluded from the study for the following reasons: four had history of coronary artery disease, one had history of corrected tetralogy of Fallot, and one due to severe aortic stenosis.

### Healthy controls

To match with PXE-patient characteristics of age, gender, and body mass index, we selected 30 volunteers with no history of cardiovascular disease or diabetes, normal blood pressure, normal cardiovascular examination, and normal resting electrocardiogram.

### Echocardiography

Images were taken in the left lateral decubitus position by means of a commercially-available VIVID 7 system (GE Healthcare, Horten, Norway) with a 2.5 MHz transducer at a depth of 14 to 16 cm. All echocardiographic data was analyzed following study termination with the GE Echopac (GE healthcare, Horten, Norway) by a single skilled interpreter blinded to the medical history. Standard data on bi-dimensional echocardiography was collected in line with the American Society of Echocardiography guidelines [9]. Left atrial diameter was measured on the parasternal long axis view, and two-dimensional linear left ventricular (LV) measurement from the parasternal view used to evaluate LV wall size. LV hypertrophy was defined by LV mass >115 g/m^2^ in men and >95 g/m^2^ in women.

### Diastolic function analysis

We performed pulsed-wave Doppler spectrograms with the area of interest set at the level of the mitral valve tips. E/A ratio was defined as the ratio of the peak early to late mitral inflow velocity. E-wave deceleration time was calculated as the time interval from the peak of E-wave to the baseline. Peak diastolic myocardial velocity (Em) was measured using pulsed-wave tissue Doppler imaging with the area of interest positioned in the mitral annulus in the lateral wall.

Diastolic function was assessed through applying the Nagueh *et al.* [10] and Kuznetsova *et al.* [11] criteria as follows: patients with E/A ratio >0.8 and E/Em ratio <12 were considered as having normal diastolic function; patients with E/A ratio <0.8 were considered as having impaired LV relaxation; patients with E/Em ratio ≥12 were considered as having elevated LV filling pressures.

### Systolic function analysis

LV ejection fraction was calculated using biplane Simpson's rule, in apical four-chamber and two-chamber views.

For the 2D-strain analysis, we recorded the apical four-, three-, and two-chamber views, along with the parasternal short axis view at papillary muscle level, in cine-loop format triggered to the QRS complex over one heart cycle with 55 to 70 frames/sec frame rates. These cine loops were then analyzed off-line on the EchoPAC system through frame-by-frame tracking of natural acoustic markers. Peak systolic longitudinal strain (LS) was recorded for each of the 16 segments, and global, basal, mid-LV, and apex LS was then calculated [12].

### Statistical analysis

All statistical tests were conducted by means of a commercially available statistical program (SPSS15, SPSS Inc., Chicago, Illinois, USA). Continuous variables were expressed as mean ± SD, categorical variables as count or absolute frequencies, and Student's t-tests (continuous variables) or chi-squared tests (categorical variables) were performed to compare LV diastolic and systolic parameters between PXE patients and healthy controls. A binomial logistic regression analysis was performed to address independent predictors for LV diastolic dysfunction in a model associating age, hypertension, current smoker, dyslipidemia, diabetes and PXE.

## Results

The 75 PXE patient and 30 matched control patient characteristics are displayed in Table 1. Age and gender were correctly matched in controls and PXE groups. The time between the diagnosis of PXE diagnosis and enrollment in the study was 18.9±13.7 years (median: 16, range: 5–31).

**Table 1:** Characteristics of the study population.

Characteristics (n)	PXE patients (75)	Controls (30)	p
Age, years	46±15	48±15	0.544
Female, n (%)	53 (71)	16 (53)	0.073
Body Mass Index, au	24.5±4.7	25.0±3.6	0.624
Dyslipidemia, n (%)	23 (28)	0 (0)	0.001
Current smoker, n (%)	20 (24.4)	0 (0)	0.001
Diabetes, n (%)	7 (8.5)	0 (0)	0.061
Hypertension, n (%)	20 (24.4)	0 (0)	0.001

Data are presented as n (%), mean ± standard deviation.

LV hypertrophy was observed in six (8%) PXE patients (three with history of hypertension) and two (7%) controls (p = 0.588).

### Diastolic function

PXE patients and controls did not differ in terms of any of the diastolic parameters tested (Table 2). Impaired relaxation with normal LV filling pressures was found in seven (9%) PXE patients and two (7%) controls (p = 0.783), as well as a slight increase in LV filling pressure in two (2.7%) other PXE patients (E/Em ratio at 12 and 16), both with history of hypertension.

**Table 2:** Echocardiographic data.

Echocardiographic data (n)	PXE patients (75)	Controls (30)	p
Heart Rate, bpm	67±9	70±12	0.179
LVEDV index, ml/m^2^	59.8±11.2	62.5±11.3	0.290
LVESV index, ml/m^2^	23.3±5.0	23.3±5.9	0.995
LVEF, %	60.8±6.0	62.9±5.1	0.117
Global longitudinal strain, %	−19.7±2.6	−20.5±2.1	0.144
Basal LS	−18.2±2.9	−17.8±2.0	0.473
Mid-LV LS	−20.2±2.4	−20.9±2.0	0.186
Apex LS	−22.2±4.5	−23.2±3.1	0.271
Left atrial diameter, mm	33.4±5.8	35.1±3.6	0.146
E wave, cm/s	82±17	82±13	0.890
E wave deceleration time, ms	191.7±55.6	190.0±35.9	0.879
A wave, cm/s	68±20	71±10	0.568
E/A ratio	1.25±0.38	1.17±0.17	0.256
Em lateral, cm/s	12±4	13±4	0.384
E/Em ratio	7.1±2.3	7.0±1.8	0.829

LVEDV, Left ventricular end diastolic volume; LVESV, Left ventricular end systolic volume; LVEF, Left ventricular ejection fraction; LS, Longitudinal strain; E, early transmitral flow velocities; A, late transmitral flow velocities; Em, septal early diastolic velocities of the mitral annulus.

Data are presented as n (%), mean ± standard deviation.

In multivariate analysis, older age was the sole independent predictor of LV diastolic dysfunction with an OR of 9.59 [2.67; 34.48].

### Systolic function

LV volumes and ejection fraction, as well as global, basal, mid-LV, and apex longitudinal strains, did not significantly differ between PXE patients and controls (Table 2).

## Discussion

This study describes the systematic assessment of cardiac function in a large cohort of asymptomatic PXE patients.

This cohort manifested no global LV systolic function impairment, in line with previous studies [4], [5]. LV longitudinal strain analysis, a sensitive tool used to detect early systolic dysfunction in preclinical disease [7], has never before been performed in the PXE setting. Our study demonstrated that longitudinal strain did not differ between PXE patients and controls.

Degenerated elastic fibers with calcification in the subendocardium have previously been observed in PXE cases [3], and one report of congestive heart failure related to restrictive cardiomyopathy has also been published [13]. In their study employing standard echocardiography and tissue Doppler imaging, Nguyen *et al.* presented seven cases of diastolic dysfunction in 19 PXE patients [5]. Campens *et al.* recently published a report involving 32 PXE patients, diastolic dysfunction was found to be associated with PXE based on significantly prolonged deceleration time and lower septal early diastolic velocities of the mitral annulus compared with controls [4]. In our larger cohort, the data did not support the theory of diastolic dysfunction being common in PXE patients. None of the diastolic parameters significantly differed between PXE patients and controls. Nevertheless, we did observe seven cases (9%) of slightly impaired relaxation and two cases (3%) of presumed LV elevated filling pressures in PXE patients, along with two cases (7%) of slightly impaired relaxation in controls. These findings did not provide strong evidence of an association between preclinical cardiac disease and PXE. Diastolic dysfunction, assessed by echocardiography, is frequently observed in the general population with a prevalence of approximately 25% [14]. The slight diastolic dysfunction we observed in a small number of patients may simply reflect the higher incidence of hypertension in PXE cases. The prevalence of hypertension in PXE has been reported ranging from 8% [1] and up to 41% of the patients [15]. One could suggest the responsibility of the increased arterial stiffness resulting from the calcification process [16].

The difference noted between PXE patients and controls in the Campens study may be due to the unexpectedly low E-wave deceleration time value (141.5 ms) observed in the control group, which was measured at 190.0 ms in our control group and approximately 200 ms in a large cohort of healthy patients of a similar age [17]. In the PXE population, very similar E-wave deceleration times were recorded in both the Campens study and our own (187.4 ms and 191.7 ms, respectively), and both within normal range [18].

Our study findings demonstrate LV systolic and diastolic dysfunction to be uncommon in the PXE setting, in line with our group's recent publication that reported very low rates of cardiac events in a prospective cohort of 67 PXE patients [6]. We can therefore conclude that asymptomatic PXE patients definitely require thorough initial cardiovascular evaluation, including echocardiography, yet echocardiographic follow-up rates should be based on symptom manifestation.

## Limitations

Although the data were obtained with one of the largest PXE cohorts published to date, the small number of old patients limited information about the onset of cardiac dysfunction with age. We previously reported heart weight and cardiomyocyte size increases in 24-month-old Abcc6 knock-out mice [6]. Nevertheless, older age was the sole independent predictor of LV diastolic dysfunction in the present cohort.

## Conclusion

This data demonstrates that preclinical cardiac dysfunction was uncommon in a large population of asymptomatic PXE patients.
